# Epidemiology and risk factors for thrombosis in children and newborns: systematic evaluation and meta-analysis

**DOI:** 10.1186/s12887-023-04122-x

**Published:** 2023-06-15

**Authors:** Shuang Song, Zhuowei Li, Guozhen Zhao, Xintong Li, Runying Wang, Bo Li, Qingquan Liu

**Affiliations:** 1 Integrative Medicine Institute, Tianjin University of Traditional Chinese Medicine, Tianjin, China; 2 Chinese Center for Disease Control and Prevention, Beijing, China; 3 Integrative Medicine Institute, Beijing University of Traditional Chinese Medicine, Beijing, China; 4 ICU, Beijing Traditional Chinese Medicine Hospital Affiliated to Capital Medical University, Beijing, China; 5 Beijing Institute of Traditional Chinese Medicine, Beijing, China

**Keywords:** Epidemiology, Risk factors, Thrombosis, Newborns, Meta-analysis

## Abstract

**Background:**

Thrombosis is a serious condition in children and neonates. However, the risk factors for thrombosis have not been conclusively determined. This study aimed to identify the risk factors for thrombosis in children and neonates in Intensive Care Unit (ICU) through a meta-analysis to better guide clinical treatment.

**Methods:**

A systematic search of electronic databases (PubMed, Embase, Cochrane Library, WOS, CNKI, Wanfang, VIP) was conducted to retrieve studies from creation on 23 May 2022. Data on the year of publication, study design, country of origin, number of patients/controls, ethnicity, and type of thrombus were extracted. The publication bias and heterogeneity between studies were assessed, and pooled odds ratios (ORs) and 95% confidence intervals (CIs) were calculated using fixed or random effects models.

**Results:**

A total of 18 studies met the inclusion criteria. The incidence of thrombosis in children was 2% per year (95% CI 1%-2%, *P* < 0.01). Infection and sepsis (OR = 1.95, *P* < 0.01), CVC (OR = 3.66, [95%CL 1.78–7.51], *P* < 0.01), mechanical ventilation (OR = 2.1, [95%CL1.47–3.01], *P* < 0.01), surgery (OR = 2.25, [95%CL1.2–4.22], *P* < 0.01), respiratory distress (OR = 1.39, [95%CL0.42–4.63], *P* < 0.01), ethnicities (OR = 0.88, [95%CL 0.79–0.98], *P* = 0.78), gestational age (OR = 1.5, [95%CL1.34–1.68], *P* = 0.65)were identified as risk factors for thrombosis.

**Conclusions:**

This meta-analysis suggests that CVC, Surgery, mechanical ventilation, Infection/sepsis, gestational age, Respiratory distress, and different ethnicities are risk factors for thrombosis in children and neonates in ICU. These findings may help clinicians to identify high-risk patients and develop appropriate prevention strategies.

**Trial registration:**

PROSPERO (CRD 42022333449).

**Supplementary Information:**

The online version contains supplementary material available at 10.1186/s12887-023-04122-x.

## Introduction

In newborns and children, thrombosis is often underdiagnosed [1]. Neonatal and pediatric arterial or venous thrombosis ranges between 2.6 and 6.4 cases per 100,000 per year [2, 3]. The incidence of thrombosis in the pediatric population is highest in newborns. Thromboses tend to occur in very sick neonates, particularly preterm neonates [4]. pediatric thrombosis has occurred more often in intensive care units(ICU) [5]. After neonatal thrombosis has received attention, case–control studies and cohort studies have gradually increased [6], but the results of different studies on the factors affecting thrombosis are different [7].

In recent years, significant progress has been in research on the influencing factors of thrombosis in children [8]. Studies have shown that several factors contribute to the incidence of thrombosis in this population, including genetic predisposition, underlying medical conditions, and environmental factors [9]. However, the level of the studies varied, and the accuracy of the individual results needs to be further determined [10, 11]. For example, the latest large cohort study found differences in risk factors for venous versus arterial thrombosis in neonates and central access device characteristics with neonatal thrombosis in ICU [8]. Whether male and female, ethnicity, antenatal maternal history, central venous catheters, catheters of different sites, or catheter length are all factors contributing to thrombosis in children and newborns is controversial [12]. And as the incidence increases, thrombosis in children is no longer rare, and its incidence in ICU needs to be further determined [13].

Currently, there are no meta-analyses of the overall factors associated with thrombosis in the ICU. The available meta-analyses of thrombosis versus catheter and thrombophilia only explored a single factor of ductal [14] or thrombophilia [15]. However, the risk factors for thrombosis have not been conclusively determined. This study aimed to identify the risk factors for thrombosis in children and neonates, allowing clinicians to develop more effective preventative and therapeutic strategies for reducing its incidence.

## Materials and methods

This systematic review and meta-analysis were conducted by the Preferred Reporting Program for Systematic Reviews and Meta-Analyses (PRISMA) statement. The study protocol was prospectively registered (PROSPERO ID: CRD 42022333449).

### Literature search and literature screening

The search time frame was for studies in each database from the creation date to 23 May 2022. Computer searches of PubMed, Embase, Cochrane Library, WOS, CNKI, Wanfang, and VIP databases extracted data on the year of publication, study design, country of origin, number of patients/controls, ethnicity, and type of thrombus. The search was conducted using Chinese and English subject terms paired with free terms, using the appropriate Boolean logical operator linkage. Literary terms include "intensive care unit ", "thrombosis in children and neonates" (under 14 years of age), "risk factors", "epidemiology", "case–control", "array research". (Data sources and search strategies are detailed in Supplementary Table 1.). The literature included the association between age, weight, ethnicity, nationality, year of publication, study design, number of patients/controls, type of thrombosis or indicators of thrombosis incidence at least one and thrombosis in children.

Two researchers independently read the titles and abstracts according to the inclusion and exclusion criteria. The irrelevant literature was excluded and extracted according to a self-designed data extraction form. Details of data extraction and quality assessment are given in Supplementary Method 1.

### Literature inclusion and exclusion criteria

Inclusion criteria:(1) Study of children aged 0–14 years and newborns in ICU; (2) The included population had thrombosis. (3) The primary outcome was the incidence of thrombosis, and the secondary outcome was the risk factors of thrombosis in neonates and children. (4) Study types include published case–control and cohort studies, blind or not;

Exclusion criteria: (1) articles such as reviews and case reports; (2) studies with incomplete data information, no corresponding outcome indicators, no extractable data, and fruitless contact with authors; (3) studies for which the full text could not be found; (4) studies with duplicate publications; (5) studies with poor statistical methods, no multi-factor regression statistical methods, and only single factor regression.

### Literature quality assessment methods

Two independent reviewers will evaluate the included literature’s quality by the Newcastle–Ottawa Scale (NOS), and any disagreements will be resolved through discussion. If still unresolved, third-party advice will be sought.

### Data extraction

Data extraction included: (1) basic information: title, first author, year of publication, study country, sample size, study design, outcomes; (2) study methodological content: study design, statistical methods, study population selection, comparability of components, outcomes; (3) epidemiological outcome indicators: age, weight, ethnicity, nationality, year of publication, study design, number of patients/controls, type of thrombus, the thrombus incidence; (4) influencing factors outcome indicators: fetal zero age, sex, a central venous catheter (CVC), infection and sepsis, mechanical ventilation, prolonged mechanical ventilation, surgery, other.

Epidemiological indicators are subject to data correction by contacting the relevant author according to the region in which the article was published. If the required data cannot be found in the published report, the corresponding author is contacted to provide the missing data of interest. Clarification was requested from the authors for parts of the article that were not specified.

### Statistical analysis

Data analysis was performed using STATA (Version 14) and the R language (4.1.1).

- (1) Selection of effect sizes. For dichotomous variables, the dominance ratio (OR) was used as the effect size; for continuous variables, continuous data were expressed as the median (minimum to maximum), and 95% confidence intervals (CI) were calculated for both. Values of P < 0.05 were considered to be statistically significant.
- (2) Evaluation of literature quality. The Newcastle–Ottawa Scale (NOS) was used to evaluate the quality of the literature. The NOS consists of 3 parts: study population selection, comparability, and exposure or outcome evaluation, with eight entries and a total score of 9. The higher the score, the better the quality.
- (3) Heterogeneity test. Inter-study heterogeneity was quantified by the I2 statistic, where I2 > 50% was evidence of substantial heterogeneity. If there was no heterogeneity, a fixed-effects model was used for analysis; if there was heterogeneity, sensitivity analysis is used to explore whether the results are robust. Sensitivity analysis uses a case-by-case rejection method. Study was excluded sequentially, and the remaining articles (n-1) were combined in a meta-analysis, and the changes in the combined results were observed to assess whether the original meta-analysis results changed significantly due to the influence of some studies.
- (4) Other analyses: the presence of publication bias was determined by a funnel plot combined with Egger's test.

## Results

### Literature screening results

**Fig. 1:**
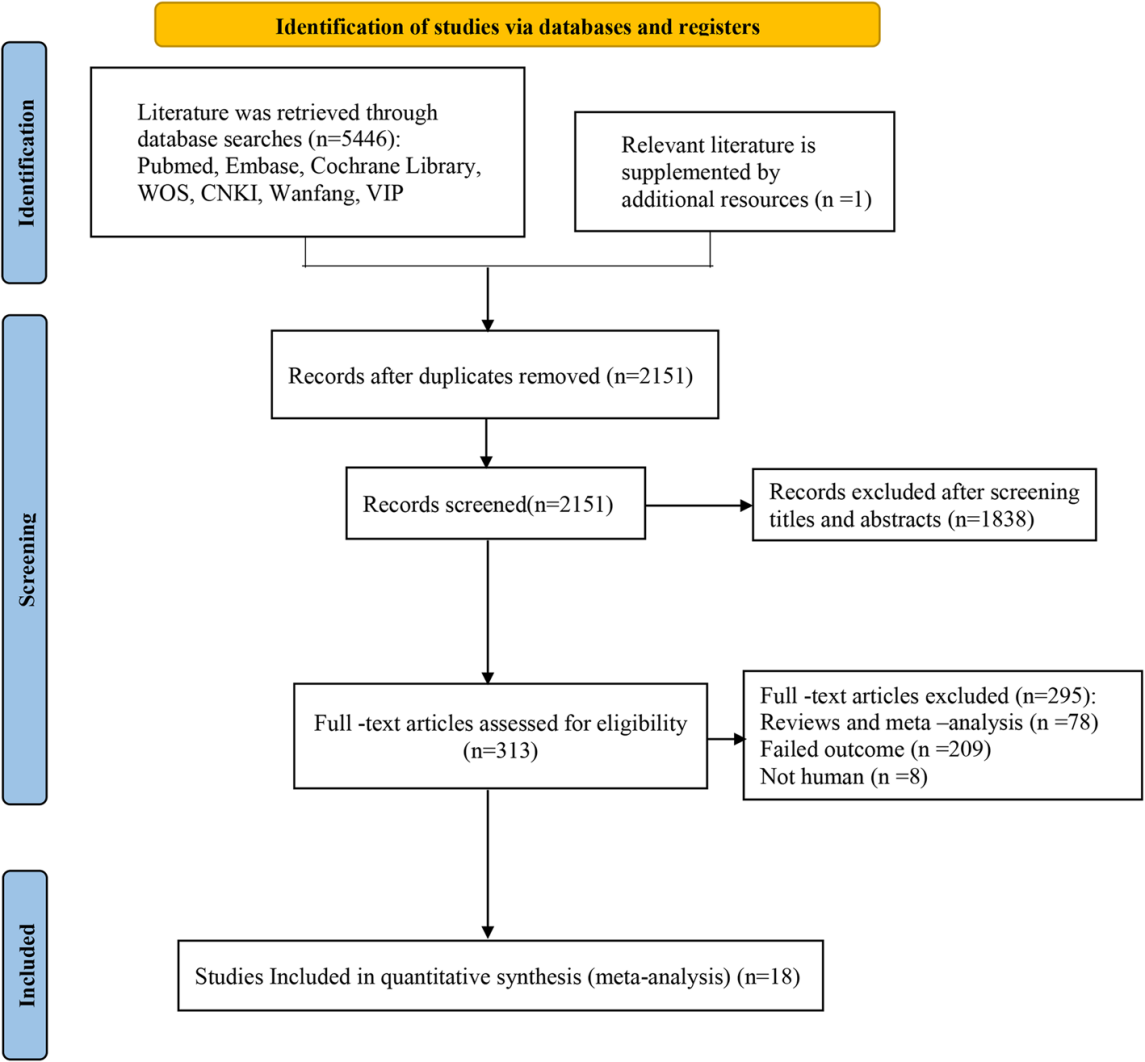
Literature search process

All literature was imported into NoteExpress software to check and exclude duplicates. The literature search initially identified 2151 non-duplicate citations. Upon title and abstract review, 1838 citations were excluded, leaving 313 articles for full-text review. Upon full-text review, 295 articles were excluded, leaving a total of 18 models for inclusion in our systematic review (Fig. 1) [16–33].

### Risk of bias in included studies

The final 18 [16–33] included studies that had 1,371,608 investigators and 12,760 pediatric thrombosis cases. Of these studies, 3239 patients with thrombosis and 1,207,450 control children were analyzed. Only one risk factor for thrombosis in children has been reported in the literature, and other cases were neonates at 22–41 weeks gestational age. 8 of the 18 studies reported on incidence; 17 reported on influencing factors. The Newcastle–Ottawa Scale (NOS) was used to evaluate the quality of the literature. The NOS consists of 3 sections: study population selection, comparability, and exposure or outcome evaluation, with a total of 8 items and a total score of 9; the higher the score, the better the quality. The quality of the included literature was rated from 6 to 8 with a mean score of (6.68 ± 0.54), and the included literature was relatively good. (Table 1, one star represents one point, the more stars after evaluation, the better the quality, preferably ten.) Table 2 illustrates the number of studies and patients included in the meta-analysis and the 95% CIs under the summary ORs and fixed or random effects models. It also shows the results of tests for heterogeneity and non-combinability. Publication bias was also calculated when > 4 studies were pooled. No significant publication bias was found in the studies except for morbidity. (Table 2).

**Table 1:** Basic information on the included literature

No	Author	Year	Country	Study design	Statistical methods	Selection	Comparability	Outcome	Score	Ending variables
1	S.Shafeek [16]	2018	Egypt	Forward-looking queues	Multiple logistic regression	✰✰✰	✰	✰✰	6	①,⑤
2	Jaffray [17]	2022	United States	Case–control	Multiple logistic regression	✰✰✰	✰✰	✰✰✰	8	②,③
3	Chojnacka [18]	2022	Poland	Case–control retrospective	Multiple logistic regression	✰✰✰	✰✰	✰✰	8	②
4	El-Naggar [19]	2020	Canada	Retrospective Matching Queue	Multiple logistic regression	✰✰✰✰	✰	✰✰	7	①
5	Bhat [20]	2022	United States	Case–control	Multiple logistic regression	✰✰✰	✰✰	✰✰✰✰	9	②,④,⑤,⑦
6	Amankwah [21]	2014	United States	Case–control	Multiple logistic regression	✰✰✰	✰✰	✰✰✰	8	③
7	Bhat [22]	2015	United States	Case–control	Multiple logistic regression	✰✰✰	✰✰	✰	8	④
8	Bhatia [23]	2022	Canada	Case–control	Multiple logistic regression	✰✰✰	✰✰	✰✰✰	8	②,⑧
9	Bhat [24]	2018	United States	Case–control	Multiple logistic regression	✰✰✰	✰✰	✰✰✰	8	②
10	Lambert [25]	2019	United States	Retrospective cohort	Multiple Cox ratios	✰✰✰	✰✰	✰✰	7	①
11	Cabannes [26]	2018	France	Forward-looking queues	Multiple logistic regression	✰✰✰	✰✰	✰✰✰	8	①,③
12	AlTassan [27]	2014	Saudi Arabia	Forward-looking queues	Multiple logistic regression	✰✰✰	✰✰	✰✰✰	8	①
13	Robinson [28]	2021	United States	Retrospective cohort	Multiple logistic regression	✰✰	✰✰	✰✰	6	①,②, ③,④, ⑤,⑥,⑦
14	Ulloa-Ricardez [29]	2016	Mexico	Case–control retrospective	Multiple logistic regression	✰✰✰	✰✰	✰✰✰	8	⑦
15	Bhatia [30]	2021	Canada	Retrospective cohort	Multiple logistic regression	✰✰✰	✰✰	✰✰	6	①
16	Sirachainan [31]	2018	Thailand	Retrospective cohort	Multiple logistic regression	✰✰✰	✰✰	✰✰	7	①
17	Giordano [32]	2018	Italy	Case–control retrospective	Multiple logistic regression	✰✰✰	✰✰	✰✰	7	⑧
18	Boo [33]	1999	Malaysia	Forward-looking observations	Multiple logistic regression	✰✰	✰✰	✰✰		③,⑥,⑧

① morbidity; ② infection and sepsis; ③ CVC; ④ mechanical ventilation; ⑤ gestational age; ⑥ ethnicity; ⑦ surgery; ⑧ respiratory distress

**Table 2:** Heterogeneity (I), publication bias in the epidemiology (incidence, infection and sepsis, CVC, mechanical ventilation, gestational age, ethnicity, surgery, respiratory distress) of thrombosis in children

Type of thrombosis (No. of Studies)	Patients/Control Subjects, n	OR/95% CI (Fixed-Effects or Random-Effects Model)	*I*^2^, %; *P*	Bias Indicator, *P*
① Incidence of disease	1,210,689, 8	0.02[0.01,0.02]	99% (*p* < 0.001)	0.032
② Infection and sepsis	1,278,550, 5	1.96[1.45–2.65]	74% (*p* = 0.04)	0.152
③ CVC	1,164,297, 6	5.75[4.32–7.67]	0% (*p* = 0.49)	0.85
④ Mechanical Ventilation	1,281,829, 3	2.23[2.00–2.49]	80% (*p* < 0.05)	-
⑤ Gestational age	1,277,757, 2	1.5[1.34–1.68]	0% (*p* = 0.65)	-
⑥ Ethnicity	1,158,854, 3	0.88[0.78–0.98]	0% (*p* = 0.78)	-
⑦ Surgery	12,777,933, 3	2.25[1.2–4.22]	98% (*p* < 0.01)	-
⑧ Respiratory distress	218/4741, 2	1.39[0.42–4.63]	84% (*p* < 0.01)	-

① morbidity; ② infection and sepsis; ③ CVC; ④ mechanical ventilation; ⑤ gestational age; ⑥ ethnicity; ⑦ surgery; ⑧ respiratory distress

### Morbidity

**Fig. 2:**
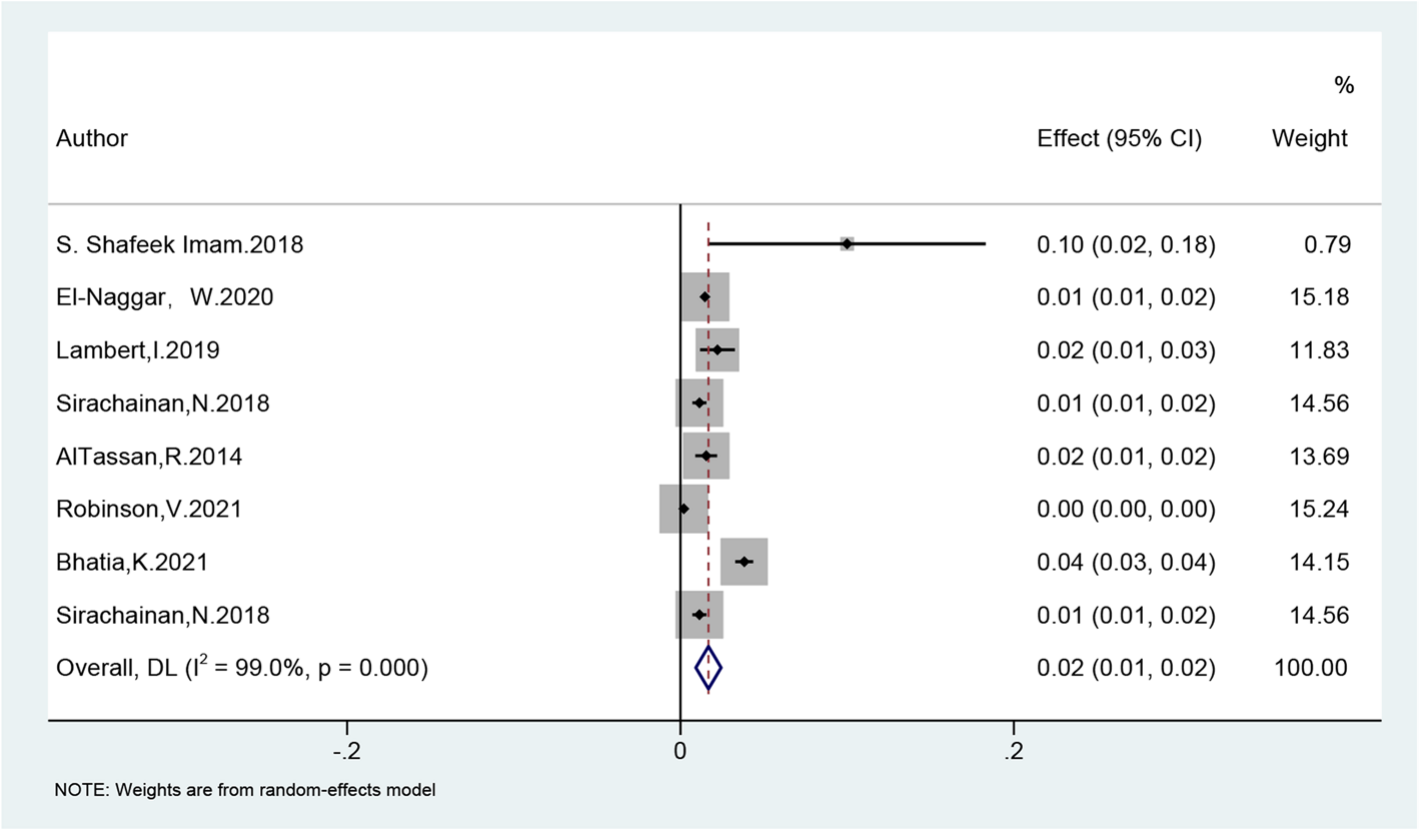
Forest plots for Meta-analysis of prevalence in the literature of thrombosis studies in children in ICUs by country

8 cohort studies [16, 19, 25–28, 30, 31] described the incidence of thrombosis. Meta-analysis showed a significant heterogeneity across studies (I^2^ = 99%, *P* < 0.01), so a random effects model was used for meta-analysis, and the incidence of thrombosis in children (per year) was 2% (95%CI (1%-2%), *P* < 0.01) (Fig. 2).

### Influencing factors for thrombosis

**Fig. 3:**
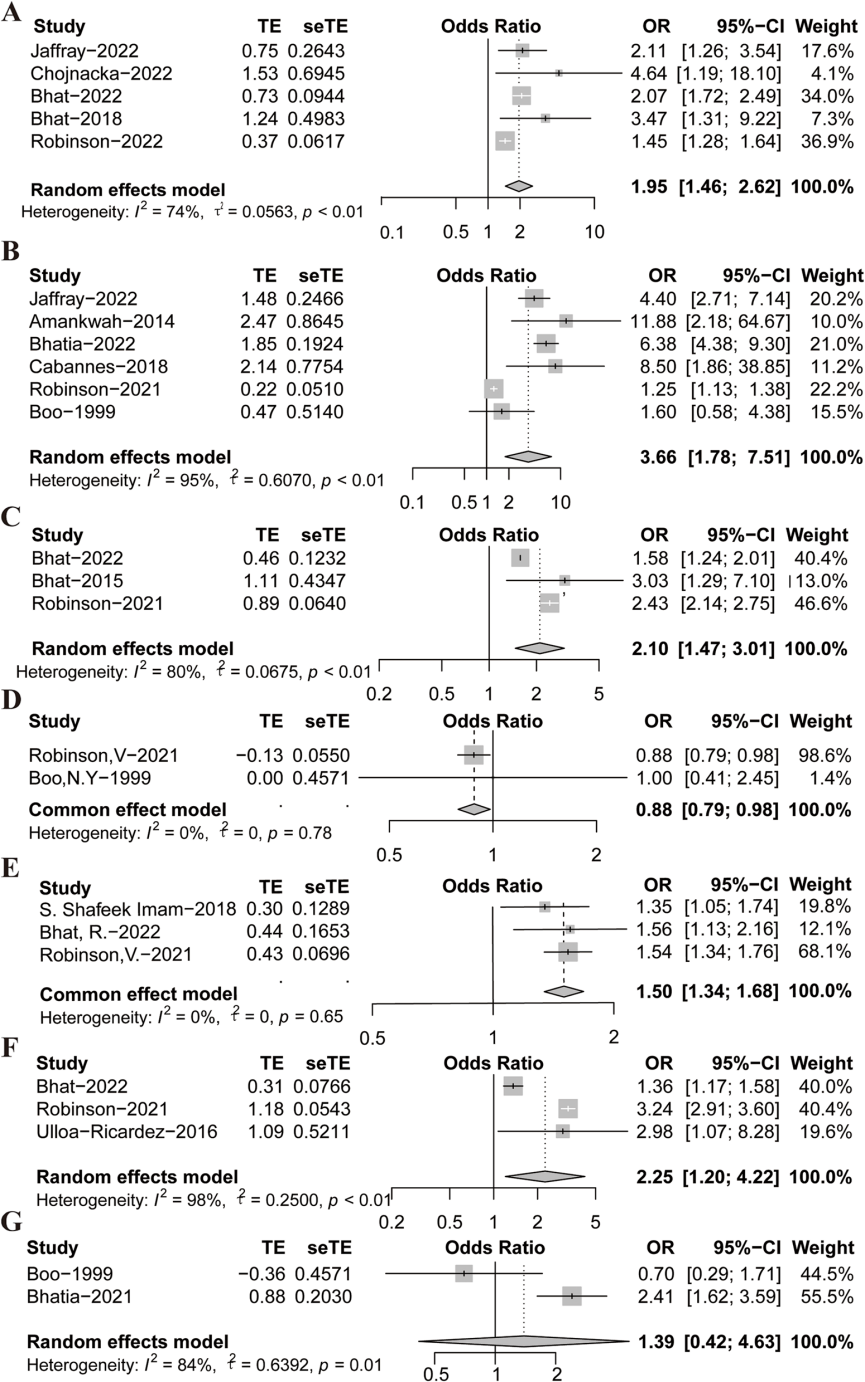
Forest plots for Meta-analysis of influencing factors in the literature on thrombosis studies in children in the ICU, Reported the effects of different influencing factors on thrombosis in children; **A **Sepsis, **B **CVC, **C **Mechanical ventilation, **D **Ethnicity, **E **Age, **F **Surgery, **G **Respiratory distress

All selected influencing factors were statistically significant. Infection, sepsis was a risk factor for thrombosis [17, 18, 20, 22, 28] (OR = 1.95, z = 0.0563, *P* < 0.01). CVC [17, 21, 23, 26, 28, 33] associated with risk of thrombosis OR = 3.66, [95%CL 1.78–7.51], z = 0.6070, *P* < 0.01; mechanical ventilation [20, 22, 28] associated with risk of thrombosis OR = 2.1, [95%CL1.47–3.01], z = 0.0675 *P* < 0.01; different ethnicities [28, 33] associated with risk of thrombosis (OR = 0.88, [95%CL 0.79–0.98], *P* = 0.78) and there was no significant heterogeneity; gestational age [16, 20, 28] associated with risk of thrombosis OR = 1.5, [95%CL1.34–1.68], *P* = 0.65. Surgery [20, 28, 29] associated with risk of thrombosis OR = 2.25, [95%CL1.2–4.22], *P* < 0.01; Respiratory distress [23, 33] associated with risk of thrombosis OR = 1.39, [95%CL0.42–4.63], *P* < 0.01. (Fig. 3 Note: Reported the effects of different influencing factors on thrombosis in children; A: sepsis, B: CVC, C: mechanical ventilation, D: ethnicity, E: age, F: surgery, G: respiratory distress.)

### Publication bias testing and sensitivity analysis

Sensitivity analysis uses “leave one out” method. Studies were excluded sequentially, and the remaining articles (n-1) were pooled for meta-analysis to observe changes in pooled results to assess whether the original meta-analysis results of thrombosis, infection and sepsis in children with high heterogeneity and CVC were significantly altered due to the impact of some studies.

**Fig. 4:**
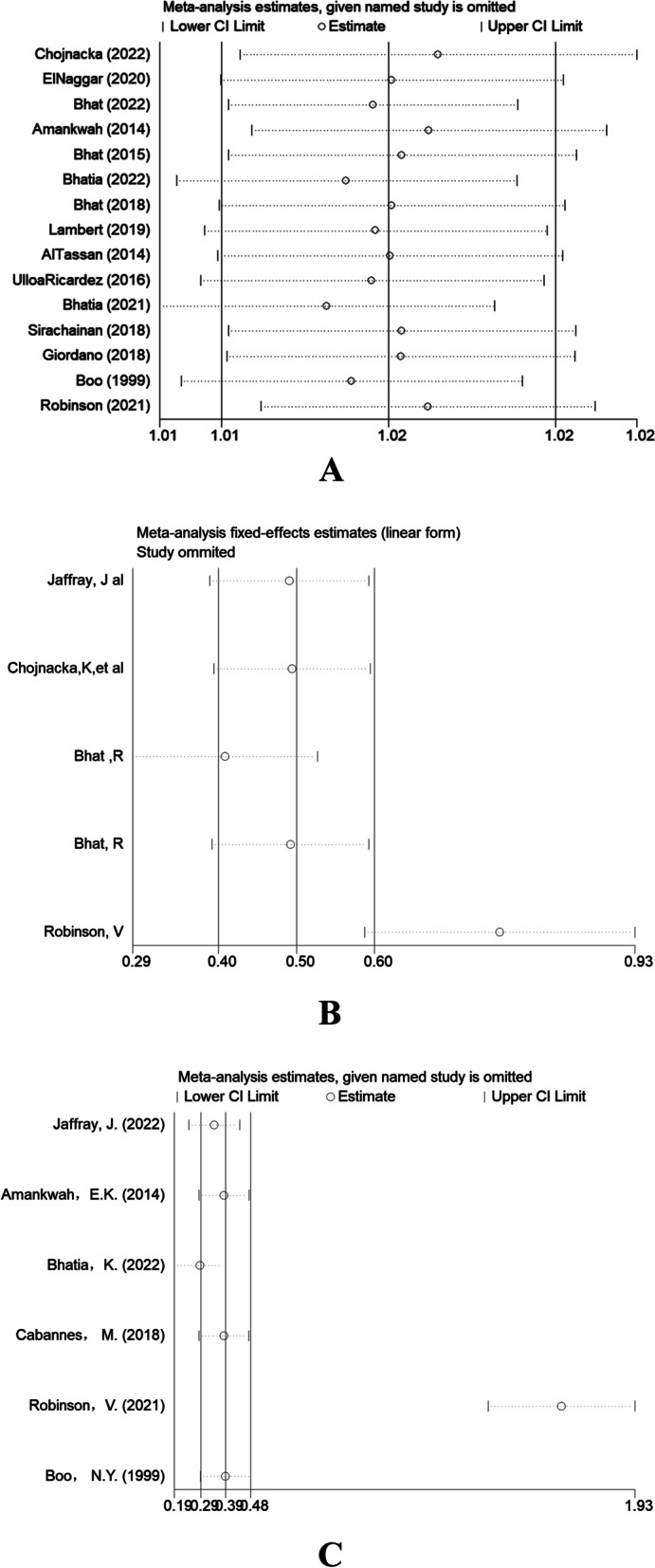
Sensitivity analysis of publication bias in children's thrombosis incidence, infection and sepsis, and CVC research literature, The OR value is the abscissa; **A **Sepsis, **B **CVC, **C **Mechanical ventilation

The Egger test for the incidence of thrombosis in children showed a publication bias in incidence (*P* = 0.032), so further testing was performed using a random effects model (log OR = 0.016, 95% CL0.012, 0.021) using a cut-and-complement method. Meta-analysis was re-run on all studies after including data from eight dummy studies (Q = 4305.860, OR1.004, [95% CI 1.000, 1.008], *P* < 0.0001). The results were not statistically significant, no reversals occurred, and the combined results were robust. (Fig. 4 note: The OR value is the abscissa; A: sepsis, B: CVC, C: mechanical ventilation.)

Infection and sepsis were funnel plot analysis among the influencing factors identified as a possible publication bias in the included literature. Further symmetry tests were performed, and there was no publication bias in this study. The CVC funnel plot was approximately balanced asymmetry. Statistical tests were insignificant (*P* = 0.152), and this study had no publication bias.

Sensitivity analysis found that among the influencing factors, except for the influencing factors of infection, sepsis, and CVC, the meta-analysis results were relatively stable. If any literature was removed, there was no significant change in heterogeneity, and the results were stable. The Robinson [28] literature on infection and sepsis had a more substantial influence on the results of the articles, so the results of Robinson [28] was removed, and the effect sizes re-combined for analysis were : Retesting revealed no significant heterogeneity in the combined infection and sepsis effect sizes (z = 11.93, *p* > 0.05).

**Fig. 5:**
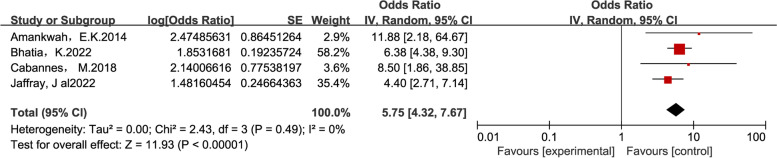
CVC influencing factors remove heterogeneity after interfering with the article

A literature-by-literature search for influencing factors CVC revealed that Robinson [28] and Boo [33] had a significant impact on the article's results, so Robinson and Boo's results were removed; the effect sizes were re-combined for analysis: retesting revealed no considerable heterogeneity in the combined CVC effect sizes (z = 11.93, OR = 5.75, *p* > 0.05) (Fig. 5).

## Discussion

We use meta-analysis method for the first time to explore the ranking of factors that influence the incidence of thrombosis in ICU. This meta-analysis summarized the incidence of thrombosis in ICU children to be approximately 2%. We found that the most significant influencing factor for thrombosis in children and newborns was CVC (OR = 3.66), followed by Surgery (OR = 2.25), mechanical ventilation (OR = 2.1), Infection/sepsis (OR = 1.95), gestational age (OR = 1.5), Respiratory distress (OR = 1.39), and different ethnicities (OR = 0.88). Next, we will discuss based on the ranking of influencing factors and analyze the incidence of thrombosis in ICU children.

The incidence rate of ICU thrombosis is 2%, which is consistent with previous studies, but higher than the incidence rate of ordinary ward thrombosis reported in other studies [34]. The incidence of neonatal thrombosis tends to increase annually and is strongly associated with the placement of catheters [35]. In the Pediatric Health Information System database, the incidence in neonates was 44 per 10,000 admissions between 2001 and 2007 [36]. It had increased to 75 in 2014 (70% increase) [36]. CVC is one of the most important influencing factors, which is consistent with previous findings of CVC on thrombosis. Previous studies found that one in five children with CVCs experienced CVC-related deep vein thrombosis (DVT) [14]. Nearly all thrombosis-related deaths are associated with CVCs and at least 85% of thrombosis are related to CVCs [37]. At present, a large-scale multi-Centre RCT experiment is needed to detect the effective prevention of CVC-induced thrombosis. Surgery is a very important influencing factor, and there is no meta-analysis to discuss the effect of surgery on pediatric thrombosis. In newborns, due to the immaturity of the coagulation and inflammatory response system, when damaged, due to its hypercoagulable state, as well as the use of adult plasma in the newborns, there is a predisposition to severe postoperative bleeding and thrombosis and systemic inflammatory response [38, 39]. Increased probability of thrombosis after organ transplantation and pediatric patients undergoing liver transplantation are inherently prone to thrombosis [40]. Mechanically ventilated patients may develop thrombosis, which is a consensus in previous studies [41]. Some studies have demonstrated a high prevalence of deep vein thrombosis in mechanically ventilated COVID-19 patients [42]. Mechanical ventilation alters the negative thoracic pressure state and decreases venous blood return and cardiac output, most likely leading to lower limb venous thrombosis [43]. In neonates, lower limb blood return is maintained mainly by negative pressure in the thorax, contraction of calf muscles and venous valves to prevent blood backflow [44]. It is advisable to strictly test coagulation and administer anticoagulation early in the postoperative period and in mechanically ventilated children or neonates to prevent thrombosis and reduce mortality [45]. For infection and sepsis, Jacques in a large prospective cohort of 2,305,380 adults undergoing surgical procedures in 374 hospitals of all types in the United States from 2005–2012 [46], found that in all surgical procedures, patients with preoperative systemic inflammatory response syndrome or any sepsis were three times more likely to have postoperative arterial or venous thrombosis. This is consistent with the findings of this paper. Respiratory distress is an important influencing factor. In child respiratory distress, research finding thrombomodulin increases, increasing the probability of thrombosis [47]. Respiratory distress syndrome is also often combined with mechanical ventilation, significantly improving the prognosis of children with respiratory disease by maintaining adequate gas exchange [42]. Gestational age is also an influence on thrombosis, and newborns born at term have a more developed coagulation system and are less likely to develop thrombosis than preterm infants [48, 49]. However, gestational age, as a factor affecting neonatal thrombosis, had less significant effects on thrombosis in children and adults [50]. Moreover, no clinical studies of gestational age as an influencing factor in thrombosis studies in children and adults. Prospectively investigated the incidence of cerebral sinus veins in infants. They found that for infants born at less than 29 weeks gestational age, systemic serial cranial US showed a very high incidence of CSVT at 4.4%. Prematurity remains the most significant risk factor for developing germinal matrix-intraventricular haemorrhage (GM-IVH) [49]. The development of the haemostatic system in neonates is an age-dependent process, with low levels of procoagulant and anticoagulant factors in preterm infants [51]. Different ethnicity also affects the incidence of neonatal thrombosis. Some studies suggest that this is because the number of NICUs varies between countries [52]. The vast differences in NICU and ICU ratios and intensive care services also lead to differences in outcomes after treatment of preterm infants, post-operative children, and other patients admitted to the ICU, resulting in different morbidity rates [2].

The study results present a baseline value for the incidence of thrombosis in children in ICU units. In addition, the included studies included a wide range of populations and different sites of thrombosis, giving incidence data for a wide range of thrombosis.

A strength of this study is the inclusion of conference articles and articles by the same authors studying different populations in different years, the uniformity of statistical methods and study methodology, the high quality of the included studies, the number of people included at 1,371,608, the separate analysis of thrombosis in children and neonates in the ICU rather than the inclusion of patients from all wards, and the relevance of the study. This paper is the first to examine all factors other than catheterization factors in thrombosis in children. Few studies have examined the factors influencing thrombosis in children and neonates in the ICU and the incidence. The literature analysis shows that although there are fewer published articles and fewer clinical trials related to thrombosis factors in the pediatric ICU, the sample size is large, the results of case studies and cohort studies are definitive, and the relevant influencing factors are uniform. At the same time, the conclusions of this paper are reliable as determined by sensitivity analysis and the cut-and-patch method and have some clinical guidance value.

There are some limitations to this study. Our search strategy may have missed some studies that were not published or not found. These cohorts may differ from the clinical characteristics of patients outside the clinical trial setting (ICU). Some important influencing factors were not analyzed because there was only one literature or no literature provided data. Therefore, we did not discuss the following influencing factors: inherited/congenital thrombotic disorders, premature neonates or not, and the influence of maternal pregnancy complications. This may ignore the role of these important factors. The Robinson [28] report, which analyzed 1,158,755 infants and was published in 2021, had a significant bias in the incidence study because it included many patients and took a retrospective cohort analysis with reliable outcomes. Finally, other possible limitations include potential interactions between thrombosis and other factors (e.g. year of publication, country), For limitations arising from the inclusion of both prospective and retrospective analyses and There is little evidence for significant effect estimate differences between Retrospective observational studies and Prospective observational studies, regardless of specific observational study design, heterogeneity, or inclusion of studies of pharmacological interventions [53].

## Conclusion

In summary, the incidence of thromboembolism using current rates is approximately 1% to 2% in the ICU. Influencing factors include CVC, Surgery, mechanical ventilation, Infection/sepsis, gestational age, Respiratory distress, and different ethnicities. Reference to the values and incidence of influencing factors can be used as a basis for ICU management and treatment of thrombosis, as well as alerting the clinic to what influencing factors need to be noted for early prophylactic treatment of different patients.

## Supplementary Information

**Additional file 1:** **Supplementary Table 1. **Data sources and search strategies. **Supplementary Method 1. **Data extraction and quality assessment.

## Data Availability

The datasets used and/or analyzed during the current study are available from the corresponding author on reasonable request.

## Abbreviations

ORs: Odds ratios
CIs: Confidence intervals
NICU: Neonatal intensive care unit
PRISMA: Preferred Reporting Program for Systematic Reviews and Meta-Analyses
NOS: Newcastle-Ottawa Scale
CVC: Central venous catheter
