# Supplementary material for: Epidemiology and risk factors for thrombosis in children and newborns: systematic evaluation and meta-analysis
Source: BMC Pediatr. 2023 Jun 15;23:292. doi: 10.1186/s12887-023-04122-x (PMC10267552; doi:10.1186/s12887-023-04122-x)
Supplement: Supplementary file 1 — Additional file 1: Supplementary Table 1. Data sources and search strategies. Supplementary Method 1. Data extraction and quality assessment. [file 12887_2023_4122_MOESM1_ESM.docx]

**Supplementary Table 1** Data sources and search strategies.

| **Databases** |  | **Search strategies** |
| --- | --- | --- |
| **PubMed**  **(n=4952)** | #1: | ((icu)[MUSH]OR(ICU))OR(intensive care unit)) |
|  | #2: | (((((((((((thrombosis)[MUSH]OR(Thromboses))OR(Thrombus))OR(Blood))OR(Clot))OR(Blood))OR(BloodClots))OR(Clot,Blood))OR(Clots,Blood)) |
|  | #3: | (((((((((((neonatal)[MUSH]OR(Infants,Newborn))OR(NewbornInfant))OR(Newborn Infants))OR(Newborns))OR(Newborn))OR(Neonate))OR(Neonates))OR(child))OR(children))OR(childhood))) |
|  | #4: | (((((epidemiology)[MUSH]OR(disease incidence))OR(incidence))OR(risk factors))OR(risk))) |
|  | #5: | #1AND#2AND#3AND#4 |
| **Web of Science**  **(n=1010)** | #1 | TS=icu or ICU or intensive care unit |
|  | #2 | TS=(thrombosis or Thromboses or Thrombus or Blood or Clot or Blood or blood clots or Clot, Blood or Clots, Blood) |
|  | #3 | #1 AND #2 |
|  | #4 | TS=(neonatal or Infants, Newborn or Newborn Infant or Newborn Infants or Newborns or Newborn or Neonate or Neonates or child or children or childhood) |
|  | #5 | TS=(epidemiology or disease incidence or incidence or risk factors or risk) |
|  | #6 | #3 AND #4 AND #5 |
| **EMBASE**  **(n=910)** | #1 | ('neonatal'::ab,ti) OR('Infants, Newborn':ab,ti OR 'Newborn Infant':ab,ti OR 'Newborn Infants':ab,ti OR 'Newborns':ab,ti OR 'Newborn':ab,ti OR 'Neonate':ab,ti OR 'Neonates':ab,ti') OR (child':ab,ti OR 'children':ab,ti OR 'childhood':ab,ti) |
|  | #2 | ('thrombosis':ab,ti )OR(Thromboses':ab,ti OR 'Thrombus':ab,ti OR 'Blood Clot':ab,ti OR 'Blood Clots':ab,ti OR 'Clot, Blood':ab,ti OR 'Clots, Blood':ab,ti ) |
|  | #3 | ('epidemiology':ab,ti )OR('risk factors':ab,ti) OR( 'disease incidence':ab,ti OR 'incidence':ab,ti OR 'risk':ab,ti)  ('(icu)':ab,ti )OR('ICU':ab,ti OR 'intensive care unit':ab,ti ) |
|  | #4 | #1 AND #2 AND #3 |
| **Cochrane Library (n=337)** |  | (((icu) OR (ICU)) OR (intensive care unit)) AND (((((((((((thrombosis) OR (Thromboses)) OR (Thrombus)) OR (Blood)) OR (Clot)) OR (Blood)) OR (Blood Clots)) OR (Clot, Blood)) OR (Clots, Blood)) AND (((((((((((neonatal) OR (Infants, Newborn)) OR (Newborn Infant)) OR (Newborn Infants)) OR (Newborns)) OR (Newborn)) OR (Neonate)) OR (Neonates)) OR (child)) OR (children)) OR (childhood))) AND (((((epidemiology) OR (disease incidence)) OR (incidence)) OR (risk factors)) OR (risk))) |
| **CNKI(n=16)**  **VIP(n=21)** **WanFang(n=0)** |  | (icu(主题词)+ICU+重症监护病房)+(血栓形成(主题词)+血液+血栓+血栓，血液+血栓，血液)*(新生儿(主题词)+新生+儿童)*(流行病学(主题词)+疾病发病率(主题词)+发病率+风险因素+风险) |

**Supplementary Method 1** Data extraction and quality assessment.

Two reviewers (GZ and XL) performed data extraction independently using a standardised predefined data extraction form, and the data were mainly extracted from published articles and supplements.

The search time frame was for studies in each database from the creation date to 23 May 2022. Computer searches of Pubmed, Embase, Cochrane Library, WOS, CNKI, Wanfang, and VIP databases extracted data on year of publication, study design, country of origin, number of patients/controls, ethnicity, and type of thrombus. The search was conducted using Chinese and English subject terms paired with free terms, using the appropriate Boolean logical operator linkage. Search terms included: study subject (Participant or Patient) P: "critical care unit" "ICU", "intensive care unit ", "thrombosis in children and neonates" (children under 14 years of age), "thrombosis", "neonatal ", "Newborns"; study outcomes (Outcome) O: "risk factors", "epidemiology" "epidemiology", "risk factors"; Study design (Study design) S: "case-control", "cohort "cohort study", "case-control", "array research". (See Annex for examples of logical operations)

Two researchers independently read the titles and abstracts according to the inclusion and exclusion criteria. The irrelevant literature was excluded and extracted according to a self-designed data extraction form.

Two independent reviewers will evaluate the included literature’s quality by the Newcastle-Ottawa Scale (NOS), and any disagreements will be resolved through discussion. If still unresolved, third-party advice will be sought.
